# The effect of hospital-based antithrombotic stewardship on adherence to anticoagulant guidelines

**DOI:** 10.1007/s11096-019-00834-2

**Published:** 2019-04-24

**Authors:** Albert R. Dreijer, Jeroen Diepstraten, Frank W. G. Leebeek, Marieke J. H. A. Kruip, Patricia M. L. A. van den Bemt

**Affiliations:** 1 Department of Hospital Pharmacy, Erasmus University Medical Center, Wytemaweg 80, 3015 CN Rotterdam, The Netherlands; 2 Department of Hospital Pharmacy, Reinier de Graaf Hospital, Delft, The Netherlands; 3 Department of Hematology, Erasmus University Medical Center, Rotterdam, The Netherlands

**Keywords:** Adherence, Anticoagulant therapy, Antithrombotic stewardship, Complex intervention, The Netherlands

## Abstract

*Background* Anticoagulant therapy is associated with a high risk of complications. Adherence to anticoagulant therapy protocols may lower this risk but adherence is often suboptimal. The introduction of a multidisciplinary antithrombotic team may improve adherence to anticoagulant guidelines among physicians. *Objective* To determine the effect of hospital-based multidisciplinary antithrombotic stewardship on adherence to anticoagulant guidelines among prescribing physicians. *Setting* This prospective non-randomised before-and-after study was conducted in patients hospitalized between October 2015 and December 2017 and treated with anticoagulant therapy. *Method* A multidisciplinary antithrombotic team focusing on education, medication reviews, drafting of local anticoagulant therapy protocols, patient counseling and medication reconciliation at admission and discharge was implemented in two Dutch hospitals. *Main outcome measure* Primary outcome was the proportion of the admitted patients in which the prescribing physician did adhere to the anticoagulant guidelines. *Results* The study comprised 1886 patients, of which 941 patients were included in the usual care period and 945 patients in the intervention period. Multivariable logistic regression analysis indicated that adherence was observed significantly more often during the intervention period (adjusted odds ratio [ORadj] 1.58, 95% confidence interval [95% CI] 1.21–2.05). Detailed analysis identified that the significantly higher overall adherence in the intervention period was attributed to dosing of LMWHs (odds ratio [OR] 1.58, 95% CI 1.16–2.14). *Conclusion* This study shows that introduction of a multidisciplinary antithrombotic stewardship leads to a significantly higher overall adherence to anticoagulant guidelines among prescribing physicians, mainly based on the improvement of dosing of low-molecular-weight-heparins.

**Electronic supplementary material:**

The online version of this article (10.1007/s11096-019-00834-2) contains supplementary material, which is available to authorized users.

## Impacts on Practice

- Multidisciplinary antithrombotic stewardship can play an important role in the improvement of adherence to anticoagulant guidelines among prescribing physicians.
- Education, medication reviews, drafting of local anticoagulant therapy protocols, patient counseling and medication reconciliation at admission and discharge are effective interventions to improve guideline adherence.

## Introduction

Anticoagulant therapy is associated with a high risk of complications [1–3]. Medication errors with anticoagulants are among the most common causes leading to harm [4, 5]. Guidelines and protocols are developed to improve prescribing quality and thus patient outcomes, and to reduce variation in clinical practice [6]. However, a discrepancy exists between recommended care and daily clinical practice [7]. In earlier studies of non-adherence to guidelines concerning proton pump inhibitor prescription in hospitalized patients who are prescribed NSAIDs, diabetes medication and dosing of medication in patients with impaired renal function, non-adherence by physicians varied between 33 and 70% [8–10].

Studies evaluating partial and/or complete compliance with the American College of Chest Physicians (ACCP) venous thromboembolism (VTE) prevention guidelines, published between 2005 and 2008, showed that compliance rates ranged from 2.8 to 84% [11]. Proietti and colleagues assessed adherence in a cohort of atrial fibrillation (AF) acutely admitted patients. They concluded that only 40.9% of the patients were treated according to the European Society of Cardiology (ESC) guideline and guideline-adherent treatment was independently associated with a significantly lower risk of all-cause and cardiovascular (CV) death [12].

Several strategies to improve guideline adherence have been described. Education programs together with computer-based clinical decision support systems showed significant improvements in adherence to guidelines for venous thromboembolism in hospitals [13]. Bos et al. [14] showed that education of hospital prescribers combined with audit and feedback by hospital pharmacists reduced physician non-adherence to guidelines covering pain management, antithrombotics, fluid and electrolyte management, application of radiographic contrast agents and surgical antibiotic prophylaxis. Furthermore, Maynard and colleagues evaluated the impact of the implementation of a multidisciplinary team on inpatient anticoagulation and management of venous thromboembolism in 189 patients with 211 identified VTE events [15]. Interventions consisted of education, computer prescriber-order-entry system (CPOE) upgrades, clinical decision support, triggered consultation, and checklists. Warfarin adjustment by protocol improved from 70 to 96% and warfarin-heparin overlap improved from 26 to 74% after the implementation of the multidisciplinary team. However, compliance to low-molecular-weight heparins (LMWHs) showed no increase and mortality and readmission rates did not change significantly. The results from previous studies showed that compliance with guidelines of different drugs varied widely and that compliance depends not only on type of drug but also on the clinical situation in which the drug is prescribed (e.g. acute care versus ambulatory care) [8–10]. Of course, depending on the situation other factors such as patient preferences may be more important than strict adherence to the guideline. Nevertheless, literature clearly shows there is room for improvement. Despite the fact that the same compliance with the prescribing guidelines for all drugs cannot be expected, there is still room for improvement. Moreover, existing anticoagulant intervention studies focused on patients treated with warfarin or low-molecular-weight-heparins (LMWHs) and do not concern patients using other vitamin K antagonists (VKAs) or direct oral anticoagulants (DOACs).

### Aim of the study

The aim of our study was to determine the effect of hospital-based multidisciplinary antithrombotic stewardship on adherence to anticoagulant guidelines by prescribing physicians.

### Ethics approval

Approval was obtained from the Medical Ethics Committee of the Erasmus University Medical Center (MEC-2015-386).

## Methods

### Study design

This study was designed as a prospective non-randomised before-and-after study, with the intervention being the implementation of a multidisciplinary antithrombotic team. Therefore a 9-month period of usual care and a 9-month intervention period were compared.

This study was a sub-study of a larger antithrombotic stewardship study (S-team study), in which the effect of a multidisciplinary antithrombotic team was evaluated on the safety and efficacy of antithrombotic therapy during hospitalization [16].

### Study setting

The study was conducted in the Erasmus University Medical Center (EMC) and the Reinier de Graaf Hospital (RdGG). The EMC is a 1320-bed University Medical Center based in Rotterdam, the Netherlands. The RdGG is a general teaching hospital located in Delft, the Netherlands, with 590 beds.

### Study population

Patients admitted to the EMC or RdGG between October 2015 and December 2017 and treated with anticoagulant therapy were eligible for inclusion. The study population consisted of patients who started on anticoagulant therapy in the hospital, patients who were already treated with anticoagulant therapy before hospitalization and patients who restarted anticoagulant therapy after a surgical or non-surgical intervention. Only the patient’s first hospital admission was included. All participants provided informed consent during hospitalization. Exclusion criteria were the following: (1) no informed consent from the patient (or the parents/guardian of the patient), (2) hospitalization for less than 24 h, (3) admission to the intensive care unit (ICU) without previous admission to a general care ward, (4) patients who received only LMWHs as thrombosis prophylaxis.

### Data collection

Data were collected from electronic patient records in the hospital information systems (HiX; Chipsoft, Amsterdam, the Netherlands and Elpado; homegrown system Erasmus University Medical Center, Rotterdam, the Netherlands) (Table S1). The bleeding risk of the surgical procedure (high, low, and clinically non-relevant bleeding risk) was defined according to the ‘Richtlijn Antithrombotisch Beleid’ (Dutch guideline on antithrombotic policy) [17]. Patient data were coded according to Dutch privacy guidelines. Data were collected during hospital stay from the day of hospitalization or from the day of discharge from the ICU to a general care ward until discharge from hospital or patient death. In patients who were initially admitted to a general care ward and subsequently transferred to the ICU, data were collected from the day of hospitalization until admission to the ICU. All data were processed with Open Clinica (Open Clinica LLC, Waltham, USA).

### Usual care

During the usual care period the normal procedures of medication surveillance by hospital pharmacists and physicians were maintained. The pharmacy software automatically checks the prescribed medication in relation to the medication record that is available within the pharmacy system and automatically generates medication surveillance alerts with a pop-up in case of drug–drug interactions, over- or underdose (dose ranges dependent on age, bodyweight and gender), duplications and contraindications. These medication surveillance alerts were easily dismissible by physicians. Furthermore, clinical rules were used in patients using DOACs or LMWHs. Clinical rules combine the renal function of the patient with the prescribed drug to assess whether dose adjustments should be made based on the renal function. A detailed description of the procedures during the usual care period can be found in the study protocol [16].

### Intervention

The previously published study protocol provides a detailed description of the antithrombotic team [16]. The intervention consisted of the implementation of a multidisciplinary antithrombotic team. The team in the University Medical Center consisted of a specialized thrombosis nurse as case manager, a hematologist, a pediatric hematologist, a hematologist (head) of the regional thrombosis service, a hospital pharmacist/clinical pharmacologist, a cardiologist, an anesthesiologist, a pulmonologist, a neurologist, a (vascular)surgeon and a quality officer. In the general hospital, the team consisted of a specialized thrombosis nurse as case manager, a hematologist, a hospital pharmacist, a cardiologist, an anesthesiologist and a clinical chemist. A neurologist, pulmonologist, pediatrician, emergency physician and (orthopedic) surgeon were added to the team when necessary. The teams focused on the following interventions:

#### Education

To increase the knowledge of antithrombotic therapy among physicians, nurses and hospital pharmacists, hospital-wide education was given.

#### Medication reviews by (hospital) pharmacists

Daily structured medication reviews were performed by the (hospital) pharmacist focused on optimizing treatment with anticoagulants. The pharmacotherapy review focused on dosing (i.e., in relation to decreased renal function, body weight and age), duplicate medication, drug–drug interactions, contraindications and perioperative bridging of anticoagulants.

#### Antithrombotic therapy guidelines

Local guidelines were drafted based on recent national guidelines and updated to ensure there was a uniform policy on antithrombotic therapy.

#### Patient counseling

The purpose of patient counseling was to provide information and education to patients with the aim of giving the patient more control and responsibility over their own health and healthcare. Such patient empowerment was performed on daily basis for each included patient.

#### Medication reconciliation

At admission, data from the patients thrombosis service regarding dosing scheme, indication for anticoagulation, type of VKA, INR measurements and the INR target range were handed over to the responsible physician. At discharge, pharmacotherapy advice from the medication review were handed over to either the thrombosis service or the general practitioner, and to the community pharmacist.

### Guidelines

Adherence to anticoagulant guidelines was assessed by using prevailing anticoagulant therapy guidelines which are implemented in the local hospital protocols. Seven guidelines were selected at which the adherence was easy to score. The guidelines focused on drug–drug interactions in patients using VKAs, dosing of LMWHs in relation to renal function and bodyweight and perioperative bridging of anticoagulants. The four separate guidelines regarding direct oral anticoagulants (drug–drug interactions in patients using DOACs, dosing of rivaroxaban versus renal function, dosing of dabigatran versus renal function and age, and dosing of apixaban versus serum creatinine, body weight and age) were clustered for the analysis into one pharmacotherapeutic DOAC measure because of the low number of DOAC users, resulting in a total of four guidelines. Table 1 shows the prevailing anticoagulant guidelines.

**Table 1:** Guidelines based on prevailing anticoagulant therapy guidelines

Pharmacotherapeutic measure	Effectuation measurement of protocol adherence	Reference
1. VKA and interacting drugs *cotrimoxazole, miconazole, fluconazole, voriconazole, amiodarone, rifampicin, rifabutin and rifaximin*	All patients with an active prescription of interacting drugs at the same time the VKA was prescribed, were checked whether the VKA or the interacting drug was discontinued and replaced by an alternative drug 24 h after the start of the combination OR whether the INR was monitored after starting the combination of the interacting drug and the VKA (within 36 h after the start of the combination with cotrimoxazole, miconazole, fluconazole, voriconazole and amiodarone AND within 5 days after the start of the combination with rifampicin, rifabutin and rifaximin)	Dutch national G-standard [18] SmPC VKA [19]
2a. DOAC and interacting drugs *ketoconazole, itraconazole, voriconazole, cyclosporin, tacrolimus, rifampicin, phenobarbital, phenytoin, carbamazepine and verapamil*	All patients with an active prescription of interacting drugs at the same time the DOAC was prescribed, were checked whether the DOAC or the interacting drug was discontinued and replaced by an alternative drug 24 h after the start of the combination. Patients treated with verapamil and dabigatran at the same time, were checked whether the dose of dabigatran was adjusted	Dutch national G-standard [18] SmPC DOAC [20]
2b. Rivaroxaban versus renal function	All patients treated with rivaroxaban, were checked whether the dose of rivaroxaban was adjusted based on the renal function	Dutch national G-standard [18] SmPC Rivaroxaban [21]
2c. Dabigatran versus renal function and age	All patients treated with dabigatran were checked whether the dose of dabigatran was adjusted based on the renal function and patient age	Dutch national G-standard [18] SmPC Dabigatran [22]
2d. Apixaban versus serum creatinine, body weight and age	All patients treated with apixaban were checked whether the dose of apixaban was adjusted based on the serum creatinine, body weight and patient age	Dutch national G-standard [18] SmPC Apixaban [23]
3. LMWH versus renal function and bodyweight	All patients treated with therapeutic doses of tinzaparin or nadroparin were checked whether the doses of the LMWHs were adjusted based on the renal function and patient body weight	EMC: Vademecum hematology [24] & Dutch national G-standard [18] RdGG: SmPC tinzaparine [25] & Dutch national G-standard [18]
4. Pre-operative INR value	All patients undergoing surgery using VKAs, were checked whether the pre-operative INR value 24 h before surgery was adequate. The cut-off pre-operative INR value was based on the bleeding risk of the surgical procedure: high (INR ≤ 1.5), low (INR ≤ 2.0), and clinically non-relevant bleeding risk (INR ≤ 3.0)	Pre-operative cut-off INR values (ACCP guideline) [26]

*VKA* Vitamin K antagonist, *DOAC* Direct Oral Anticoagulant, *LMWH* Low Molecular Weight Heparin, *SmPC* Summary of Product Characteristics, *EMC* Erasmus University Medical Center, *RdGG* Reinier de Graaf Hospital, *INR* International Normalized Ratio, *ACCP* American College of Chest Physicians

### Outcome measures

Primary outcome was the proportion of the admitted patients in which the prescribing physician adhered to one or more of the anticoagulant guidelines (the total number of admitted patients was included as denominator). Secondary outcome was the proportion of the prescriptions in which the prescribing physician adhered to each of the four anticoagulant guidelines (for the prescribed anticoagulant(s) each patient was on, the total number of applicable guidelines and opportunities for adherence was calculated and included in the denominator).

### Sample size

This study has been powered on the outcome measure of the S-team study, in which the effect of a multidisciplinary antithrombotic team on the safety and efficacy regarding antithrombotic therapy during hospitalization is studied [16]. With a type 1 error of 0.05, power of 80%, the required sample size was 917 patients in the usual care period and 917 patients in the intervention period. In order to account for drop-outs, 1900 patients were included.

### Data analysis

All data were analyzed with IBM SPSS version 21.0 (IBM Software, New York, USA). All continuous variables were tested for normality with the Shapiro–Wilk test. Non-normal variables were expressed as medians and interquartile ranges (IQR) and differences between groups tested with the Mann–Whitney *U* test. Categorical variables were presented as percentages and tested for statistical significance between groups using the Chi square test. *P* < 0.05 was considered to be statistically significant. Odds ratios (OR) and 95% confidence intervals (95% CI) for each of the four anticoagulant guidelines were obtained by logistic regression analysis, with the time period (intervention period versus usual care period) as primary variable. In order to adjust for possible predictors, multivariable logistic regression analysis was performed. The following possible predictors were initially entered into the model: age, length of hospitalization, hospital type, surgery and treatment with VKAs, DOACs or LMWHs. Variables that changed the beta-coefficient with more than 10% were retained in the model. Adjusted odds ratios (ORadj) and 95% confidence intervals (95% CI) were reported.

## Results

### Study population

**Fig. 1:**
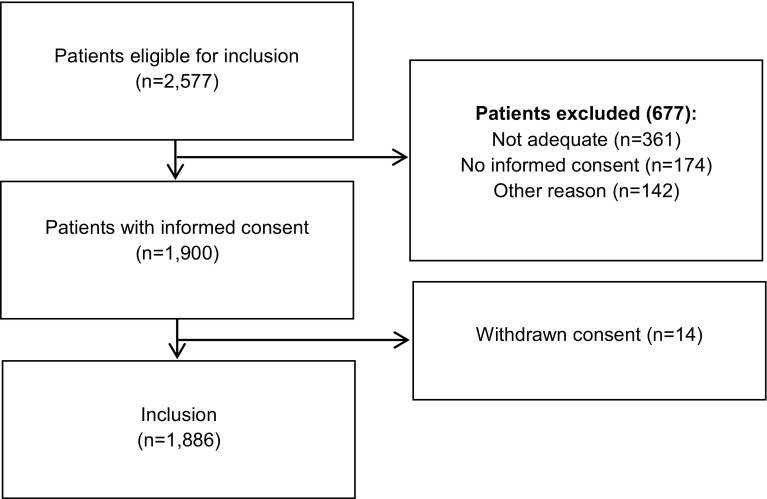
Study flow

During the study period 2577 patients were eligible for inclusion. In 677 patients, at least one reason for exclusion was present. Fourteen patients withdrew their consent after signing the informed consent due to medical reasons. Thus, in total 1886 patients were included in our analysis, which included 941 patients in the usual care period and 945 patients in the intervention period (Fig. 1). Characteristics of the included patients are presented in Table 2. Of these, the majority in both groups were male and the median age was 69 years. There were no differences between the two groups in gender, age, prior thrombotic event, hospital type, weight, renal function and high and low bleeding risk of the surgical procedure (in cases where the patients had to undergo surgery).

**Table 2:** Baseline characteristics of the patients

Characteristic	Usual care period (*n *= 941)	Intervention period (*n *= 945)	*p* value
Male gender	562 (59.7)	578 (61.2)	0.522
Age, years	69 [59–77]	69 [59–77]	0.665
Length of hospitalization, days	8 [5–14]	7 [3–13]	**<** **0.001**
Prior bleeding	198 (21.0)	269 (28.5)	**<** **0.001**
Prior thrombotic event	448 (47.6)	461 (48.8)	0.610
Hospital type, University Medical Center	472 (50.2)	472 (49.4)	0.927
Weight	80 [70–91]	80 [70–93]	0.177
e-GFR, ≤ 50 ml/min/1.73 m^2^	301 (33.0)	266 (30.1)	0.189
Surgery	340 (36.1)	330 (34.9)	0.583
*Bleeding risk surgical procedure*			
High bleeding risk	243 (25.8)	212 (22.4)	0.085
Low bleeding risk	57 (6.1)	62 (6.6)	0.653
Clinically non-relevant bleeding risk	40 (4.3)	60 (6.3)	**0.032**
Type of anticoagulant therapy*			
Vitamin K antagonist	646 (68.7)	553 (58.5)	**<** **0.001**
Direct oral anticoagulant	80 (8.5)	263 (27.8)	**<** **0.001**
Low-molecular-weight-heparin	488 (51.9)	423 (44.8)	**0.002**

Figures in bold are statistically significant

Results are presented as median [interquartile range] or as number of patients (%) for non-continues data. N, number of patients at risk; *e*-*GFR* estimated glomerular filtration rate

*Patients can use multiple anticoagulants during hospitalization

Patients included in the intervention period had a shorter hospital stay (*p* < 0.001), had more prior bleeding events (*p* < 0.001) and a larger number of patients had a surgical procedure with a clinically non-relevant bleeding risk (*p* = 0.032). The use of VKAs (*p* < 0.001) and LMWHs (*p* = 0.002) was less in patients in the intervention group but the use of DOACs was higher (*p* < 0.001).

### Adherence to anticoagulant guidelines

Table 3 shows the proportions of the admitted patients in which the prescribing physician adhered to one or more of the anticoagulant guidelines. Logistic regression analysis revealed that the overall adherence was significantly higher in the intervention period [75.3% (497/660)] compared to the usual care period [63.4% (395/623)] (odds ratio [OR] 1.76, 95% confidence interval [95% CI] 1.38–2.24). After adjustment for the possible predictors (i.e. age, length of hospitalization, hospital type, surgery and treatment with VKAs, DOACs or LMWHs), the adjusted OR was 1.58 (95% CI 1.21–2.05). As shown in Table 3, the significantly higher overall adherence in the intervention period was attributed to dosing of LMWHs in relation to renal function and bodyweight. The odds ratio was 1.58 (95% CI 1.16–2.14). The other guidelines (drug–drug interactions in patients using VKAs, perioperative bridging of anticoagulants and dosing of DOACs) showed no significant differences between the usual care period and intervention period.

**Table 3:** Adherence of prescribing physicians to guidelines based on prevailing anticoagulant therapy protocols

	Usual care period (*n *= 941)	Intervention period (*n *= 945)	OR [95% CI]
	Adherence	Adherence	
1. VKA and interacting drugs	103/111 (92.8%)	74/81 (91.4%)	0.82 [0.29–2.36]
2. DOAC and interacting drugs, renal function, age and body weight	69/80 (86.3%)	228/263 (86.7%)	1.04 [0.50–2.15]
3. LMWH versus renal function and bodyweight	217/393 (55.2%)	204/309 (66.0%)	**1.58 [1.16**–**2.14]**
4. Pre-operative INR value	180/227 (79.3%)	151/181 (83.4%)	1.31 [0.80–2.18]
Overall adherence	395/623 (63.4%)	497/660 (75.3%)	**1.76 [1.38**–**2.24]**
			**1.58**^**a**^**[1.21**–**2.05]**

Figures in bold are statistically significant

*OR* odds ratio, *95% CI* 95% confidence interval, *VKA* Vitamin K antagonist, *DOAC* Direct Oral Anticoagulant, *LMWH* Low Molecular Weight Heparin, *INR* International Normalized Ratio

^a^OR, adjusted for predictors (age, length of hospitalization, hospital type, surgery and treatment with VKAs, DOACs or LMWHs)

The proportions of the prescriptions in which the prescribing physician adhered to each of four anticoagulant guidelines occurred in 569 out of 811 (70.2%) prescriptions in the usual care period and in 657 out of 834 (78.8%) prescriptions in the intervention period. After adjustment for the same possible predictors, the adjusted odds ratio was 1.42 (95% CI 1.12–1.80).

## Discussion

The overall adherence to anticoagulant guidelines was significantly higher after the implementation of a multidisciplinary antithrombotic team focusing on education, medication reviews, drafting of local anticoagulant therapy protocols, patient counseling and medication reconciliation. The significantly higher overall adherence in the intervention period can be attributed to the improvement of dosing of LMWHs in relation to renal function and bodyweight.

Earlier multifaceted intervention studies also showed a positive impact on guideline and protocol adherence. Maynard et al. [15] revealed that implementation of a multidisciplinary team, focusing on patients with identified VTE events and treated with warfarin or LMWHs led to improved inpatient anticoagulation and management of venous thromboembolism. Bos et al. introduced an educational program for prescribers in the hospital combined with audit and feedback by the hospital pharmacist. This led to a significant decrease in non-adherence from 30.5 to 21.8% of prescribing physicians to key pharmacotherapeutic guidelines, such as gastric protection in case of use of NSAID in hospitalized surgical patients and perioperative bridging of antithrombotics [14]. Other multifaceted intervention studies focusing on antibiotics found an increase in the rate of guideline adherence of antibiotic prescription [27, 28]. The hypothesis that a multifaceted approach is the most effective method to improve protocol adherence is supported by a previous study of Worel et al. [11] who described that lack of audit tools and feedback systems and the presence of an abundance of guidelines with conflicting recommendations result in lack of guideline adherence. Furthermore, passive dissemination of guidelines alone is often insufficient to have a positive impact on guideline adherence [13]. This study shows that the implementation of a multidisciplinary antithrombotic team leads to a significant increase in adherence to anticoagulant guidelines, specifically dosing of LMWHs. The improvement was obtained on top of other measures as medication surveillance by hospital pharmacists and clinical rules, which were part of usual care. Furthermore, active strategies such as education and medication reviews are needed to increase the knowledge and skills of prescribing physicians and thereby improve the adherence of guidelines. Comparing the different intervention studies on protocol adherence with each other is difficult given that the interventions in the various studies differ from each other. Moreover, this study focused on anticoagulant guidelines including patients treated with VKAs and DOACs while other studies focused mainly on patients treated with warfarin or LMWHs for specific indications, such as VTE. The significant increase in the number of DOAC users during the intervention period compared to the usual care period may be explained with as in 2016 (at the time of the intervention period) DOACs have been recommended as the first choice treatment of VTE [29].

The majority of overall adherence to anticoagulant guidelines was mainly caused by the improvement of dosing of LMWHs (OR 1.58 [95% CI 1.16–2.14]). Slikkerveer et al. [30] found that most prescribing errors with LMWH treatment included overdosages and underdosages that were not correctly adjusted to body weight or renal function. The significantly higher adherence to dosing of LMWH therapy in the intervention period may be explained by the fact that during the intervention period medication reviews were performed by hospital pharmacists with attention paid to both bodyweight and renal function in relation to the dose of LMWHs. This differs from the usual care period where attention was only paid to the renal function in relation to dosing of LMWHs. Focusing on both body weight and renal function may have led to the improvement of dosing of LMWHs among prescribing physicians. LMWHs are one of the most frequently therapeutically prescribed anticoagulants in hospitalized patients. Besides, as dosing is based on both bodyweight and renal function, prescribing errors occur frequently. This may have contributed to the fact that the greatest effect of the hospital-based multidisciplinary antithrombotic stewardship was seen on dosing of LMWHs. Guidelines concerning drug–drug interactions in patients using VKAs and DOACs, perioperative bridging of anticoagulants and dosing of DOACs in relation to renal function, age and bodyweight showed no significant association in adherence of prescribing physicians after the implementation of a multidisciplinary antithrombotic team. A possible explanation is that during the usual care period the pharmacy software automatically checked the prescribed medication in relation to the medication record that was available within the pharmacy system and automatically generated medication surveillance and signals in case of interactions, overdose, duplications and contraindications. In addition, the pre-operative INR value before surgery was already closely monitored by the physician during the usual care period. Despite the significant increase in adherence to anticoagulant guidelines in this study, 24.7% of the prescribing physicians were non-adherent to the anticoagulant guidelines after implementation of the multidisciplinary antithrombotic team. Although this study showed that the implementation of a multidisciplinary antithrombotic team leaded to a significant increase in adherence to anticoagulant guidelines, there still may be room for improvement.

### Strengths and limitations

To our knowledge, this is the first study describing the effect of hospital-based multidisciplinary antithrombotic stewardship on the adherence to anticoagulant guidelines among prescribing physicians. Furthermore, the study was performed in two different types of hospitals, a University Medical Center and a general teaching hospital, which increases the generalizability of our findings. Another strength of this study is the multifaceted approach which combines different interventions to improve the adherence to anticoagulant guidelines.

This study has several limitations. First, seven guidelines derived from several anticoagulant therapy protocols were selected. This is a limited set of anticoagulant guidelines and may not be generalizable to all anticoagulant protocols. A second limitation is that the cost-effectiveness of the intervention has not been analyzed. Additional costs were incurred by performing medication reviews, which were conducted by the hospital pharmacist. Furthermore, drafting of local protocols and education to physicians and nurses were performed by healthcare providers, such as a specialized thrombosis nurse, a hematologist, a hospital pharmacist, and a cardiologist. Third, logistic regression analysis doesn’t take into consideration any clustering within prescriber (e.g. surgical versus medical). Fourth, this study is a prospective non-randomised before-and-after study, without a retrospective control group. Improvements may already have been implemented during the usual care period. Finally, the intervention is multifaceted making it difficult to say which specific intervention (e.g. medication reviews) has been of the greatest influence on improvement of anticoagulant therapy protocol adherence among prescribing physicians.

## Conclusion

This study showed that introduction of hospital-based multidisciplinary antithrombotic stewardship resulted in a significantly higher overall adherence to anticoagulant guidelines among prescribing physicians, mainly based on the improvement of correct dosing of low-molecular-weight-heparins. Future studies should focus whether higher adherence to anticoagulant guidelines contributes to improvement in clinical outcomes.

## Electronic supplementary material

Below is the link to the electronic supplementary material.

Supplementary material 1 (DOCX 13 kb)
