# Supplementary material for: The effect of hospital-based antithrombotic stewardship on adherence to anticoagulant guidelines
Source: Int J Clin Pharm. 2019 Apr 24;41(3):691–9. doi: 10.1007/s11096-019-00834-2 (PMC6554262; doi:10.1007/s11096-019-00834-2)
Supplement: Supplementary file 1 — Supplementary material 1 (DOCX 13 kb) [file 11096_2019_834_MOESM1_ESM.docx]

**Table S1** Data collection

| **Part** | **Data content** |
| --- | --- |
| Patient data | Patient ID |
|  | Date of birth |
|  | Gender |
|  | Weight on the day of hospitalization |
|  | Date of hospitalization |
|  | Date of hospital discharge |
|  | Type of hospital (University Medical Center/general teaching hospital) |
|  | Bleeding in history (yes/no) |
|  | Thrombotic event in history (yes/no) |
| Interventions | Surgical procedure (yes/no) |
|  | If surgical procedure, bleeding risk of the surgical procedure (high, low, or clinically non-relevant) [17] |
| Medication data | Type of anticoagulant therapy |
|  | - Vitamin K antagonist |
|  | - Direct oral anticoagulant |
|  | - Low-molecular-weight-heparin |
| Clinical chemistry data | Laboratory values |
|  | - e-GFR (ml/min/1.73m^2^) on the day of hospitalization |
|  | - INR |

*e-GFR* estimated glomerular filtration rate, *INR* International Normalized Ratio
